# AI tool could predict heart attack 10 years ahead: Oxford study

**Published:** 2023

Artificial intelligence (AI) could help predict if a person is likely to have a heart attack up to 10 years before it happens, suggest British scientists, after eight years of research found that a specifically designed tool could prevent thousands of heart attack deaths if it were rolled out. The study by the University of Oxford looked at ways to improve the accuracy of cardiac CT scans, used to detect any blockages or narrowing in the arteries, using AI, and determined that the tool could accurately predict heart attacks, reports The Independent.

Professor Charalambos Antoniades, chairman of cardiovascular medicine at the British Heart Foundation (BHF) and director of the acute multidisciplinary imaging and interventional centre at the University of Oxford, said the findings revealed that some patients who go to hospital with chest pain, and are then sent back home, have a high risk of having a heart attack in the next decade, even without any sign of disease in their arteries.

‘We demonstrated that providing an accurate picture of risk to clinicians can alter, and potentially improve, the course of treatment for many heart patients. If this AI tool were implemented across the NHS, it would help prevent thousands of avoidable deaths from heart attacks every year in the UK.’

The British Government recently announced a ￡21m pot for which NHS trusts can apply, to pay for AI tools, including those used for medical imaging and to help with treatment decision-making. According to the BHF, which funded the research, about 350 000 people in the UK have cardiac scans each year. However, many patients go on to die of heart attacks due to the failure in detecting small, undetectable narrowings in the heart.

The researchers analysed the data of more than 40 000 patients undergoing routine scans at eight UK hospitals. The AI tool was tested on a further 3 393 patients over almost eight years and found to accurately predict their risk of a heart attack. Those whose results showed ‘significant’ narrowing of the arteries were more likely to have a serious heart attack, but twice as many patients with no significant narrowings also went on to have heart attacks, which were sometimes fatal.

The team developed an AI programme that was trained using information on changes in the fat around inflamed arteries, which can signify the risk of a heart attack. Professor Sir Nilesh Samani, medical director at the BHF, said the research ‘shows the valuable role AI-based technology can play’ in identifying those most at risk of future heart attacks. 
